# Psychological interventions for depression and anxiety: a systematic review and meta-analysis of Iranian chronic pain trials

**DOI:** 10.34172/hpp.2020.31

**Published:** 2020-07-12

**Authors:** Gholamreza Jandaghi, Manijeh Firoozi, Ali Zia-Tohidi

**Affiliations:** ^1^Department of Management, Faculty of Management and Accounting, Farabi Campus, University of Tehran, Qom; ^2^Department of Psychology, Faculty of Psychology and Educational Sciences, University of Tehran, Tehran, Iran; ^3^Faculty of Management and Accounting, Farabi Campus, University of Tehran, Qom

**Keywords:** Depression, Anxiety, Chronic pain, Psychotherapy, Meta-analysis, Iran

## Abstract

**Background:** Chronic pain is commonly associated with anxiety and depression, making it more challenging to be managed. Psychological interventions are suggested for such complicated issues which are well evident in the United States and Europe. However, generalizing the evidence to Iranian population – as a Middle Eastern society – might be questionable. We aimed to synthesize our evidence on the effectiveness of these interventions among Iranian populations.

**Methods:** This was a systematic review and meta-analysis. Persian and English literature were searched through Iran-doc, Elm-net, and PubMed until March 2019 using the following terms (or its Persian synonyms): chronic pain; persistent pain; chronic fatigue; fibromyalgia; neuropath*; LBP; irritable bowel; CFS; psycho*; cogniti*; acceptance; meaning; mindfulness; relaxation; biopsychosocial; rehabilitation; educat*. Eligible trials were randomized trials that evaluated the effectiveness of psychological interventions on Iranian adults with chronic pain. No setting restriction was considered. Risk of bias for each trial was assessed, and the random-effect model was used to pool summary effect across trials.

**Results:** In all 30 eligible RCTs, the risk of bias for randomization was low except for one study. The pooled standardized mean difference (SMD) for depression and anxiety were 1.33 (95%CI: -1.42 to -0.68) and 1.25 (95% CI: -1.55 to -0.96), respectively.

**Conclusion:** This study suggests that psychological interventions are highly effective in reducing depression and anxiety in Iranian patients with chronic pain, compared to what observed in the U.S. and European studies. However, there are still some methodological issues to be addressed. Future research should focus on high-quality trials with considerations on the methodological issues reported in the present study.

## Introduction

Treating or well managing chronic pain (CP) has been considered as an unresolved issue for decades. Various medical and psychological treatments are shown to be slightly effective in relieving pain.^1^ On the other hand, CP commonly co-occurs with anxiety and depression,^2-4^ which adds more complications to the health status of the patients. According to a country-level survey in Iran, depression is the leading and the second cause of years lost due to disability in women and men, respectively.^5^ The issue becomes more dramatic when we realize that low back pain is the other leading cause (first cause in men and the second in women). Beside depression and anxiety as two major risk factors for suicide ideation and attempt,^6^ CP is also considered as one of the main risk factors for suicide, as reported by the World Health Organization (WHO).^7^

The clinical recommendations suggest that when these conditions occur concomitantly, they should be treated concurrently^8^; as CP has complicated relationships with anxiety and depression which necessitate simultaneous treatment:

Depression and pain share neurological pathways as well as psychological factors (e.g., catastrophizing, feeling of loss, behavioral avoidance), and respond to same treatments, both pharmacological (e.g., TCAs, SSRIs) and psychological (e.g., CBT).^9^

Evidence suggests that anxiety and depression could exacerbate pain intensity, facilitate the transition of the acute to CP,^10^ or even have a causal effect on the onset of pain, especially in the case of anxiety. In fact, there is evidence that in most cases of co-occurrence, anxiety precedes the onset of CP, unlike depression which usually occurs after the development of CP.^11^

There is longitudinal evidence that high level of pain interference at baseline could result in poor treatment outcomes on anxiety and depression.^12,13^

Improvement in depression and anxiety during treatment could improve pain outcomes, such as pain intensity/interference and disability, as well.^14^

Due to these interactions, and more generally due to the biopsychosocial nature of CP, related organizations in the United States have recently recommended a multidimensional framework for the diagnosis of CP conditions that incorporate social and psychological aspects of the issue —^10,15^ analogous to the multiaxial framework used in DSM-3 and 4.

To address anxiety and depression in patients with CP, researchers worldwide have conducted hundreds of RCTs (especially on psychological treatments as the interest of this research­), which has been summarized in numerous reviews and meta-analyses.^16-21^ However, the evidence is mainly based on the trials conducted in the United States and Western European countries, and though it might not be generalizable to other regions around the globe, especially to the regions with considerable cultural differences–such as the Middle East. In Hilton et al,^20^ 30 out of all included trials were conducted in the United States and Europe, and only 4 were performed in the Middle East; and In Bernardy et al,^21^ 28 out of all total 29 included trials were conducted either in the United States or Europe.

### Culture, chronic pain, and psychotherapy

Our skepticism about the generalizability of the American and European trials to a Middle Eastern population such as Iranian community is based on the fact that pain is a biopsychosocial phenomenon.^10,22,23^ It acts and responds differently in various cultural contexts. Patients’ beliefs about pain and its consequences, their behavioral response to pain, as well as their expectations, views, reactions, and perceived supports could change the way that pain affects them. (for a review on cultural differences in pain perception see Pillay et al^24^).

On the other hand, almost all well-known psychotherapies have been developed in American and European cultures, by theoreticians who grew within those environments, and through working with subjects from those cultures. Such theories may not fit well to the situations happened in other cultures.^25^

Also, concerning CP treatments, the main issue in most psychological interventions for CP is probably coping with pain (stress), which has a different pattern across various cultures. For instance, there is evidence that people from collectivistic cultures use more emotion-focused strategies than they do in individualistic cultures. Also, some coping strategies are helpful in some cultures and not in others.^26,27^

Our objective in this study was to evaluate and synthesize Iranian randomized clinical trials that investigated the efficacy of psychological interventions on anxiety and depression in adults with CP compared to usual care or waitlist. Then, we compared our results with other meta-analytical results, and tried to stress the differences between them, not only concerning the effect sizes (ESs) but also other aspects of trials, such as methodology.

## Materials and Methods

This was a systematic review and meta-analysis, which was reported based on the PRISMA guideline.

### Eligibility criteria

*Participants and settings*

Eligible participants were Iranian adults diagnosed with CP (defined as the pain that lasts for more than three months). Studies on the patients with chronic cancer pain and CP due to multiple sclerosis (As both diseases have a high level of effect on anxiety and depression, regardless of pain) were excluded. Also, the trials with less than ten participants per group were excluded. No setting restriction was applied.

*Interventions*

All psychological interventions with all formats of delivery (e.g., individual therapy, group therapy) were eligible. To reduce heterogeneity, the interventions with merely medical education and the intervention courses with less than four treatment sessions were excluded.

*Type of control*

Control groups included treatment as usual (TAU; or usual care), waitlist and psychological placebo. Accordingly, trials with other active controls were excluded (i.e., the trials within which two psychological approaches were compared).

*Outcome measures*

Studies that reported outcomes on depression or anxiety were included.

*Study design*

Only RCTs that reported as either journal articles or theses were included.

### Information sources, search strategy, and study selection

**Figure 1:**
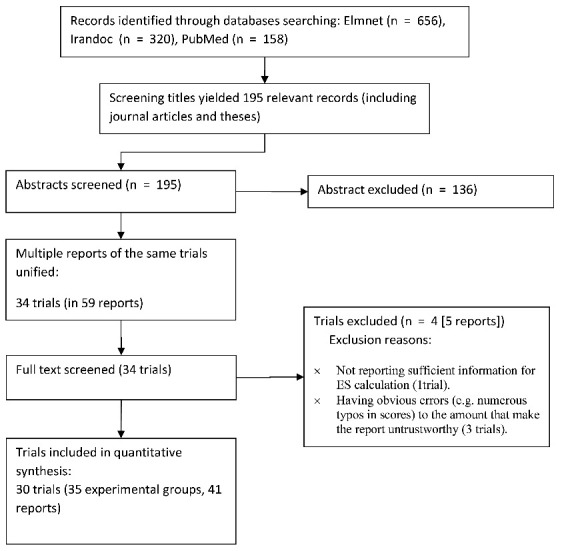
Flow diagram of study selection process.

We searched Iran-doc, Elm-net, and PubMed (until March 2019) for eligible trials using the following terms (or its Persian synonyms): chronic pain; persistent pain; chronic fatigue; fibromyalgia; neuropath*; LBP; irritable bowel; CFS; psycho*; cogniti*; acceptance; meaning; mindfulness; relaxation; biopsychosocial; rehabilitation; educat*. As such a comprehensive search strategy in PubMed resulted in about 35 000 records of which only a small proportion conducted in Iran we imposed the following additional terms: Iran; Iranian; or Persian. Titles, abstracts, and then the whole texts were screened (flow diagram of selection process presented in Figure 1).

### Assessing risk of bias in individual trials

We assessed the risk of bias at the study level, based on the Cochrane collaboration guidelines in four domains: sequence generation, allocation concealment, incomplete outcome data, and selective outcome reporting.^28^ Blindness was not assessed in this study, because blinding the therapists and the patients are usually not applicable in psychological treatments. Also, in all included studies data were collected through self-report. So, blinding the outcome assessors was inapplicable.

### Data preparation and synthesis methods

*Calculating within study effect sizes*

We used standardized mean difference (SMD) to measure ES, as the unstandardized mean difference was not appropriate due to different measurement scales across the trials. To do so, we adjusted the baseline scores, as the most of included trials had small samples, and in the studies with small sample size baseline differences could substantially affect the results. The preferred approach to account for baseline scores is the use of information from analysis of covariance applied to estimate adjusted ESs.^29,30^ However, only a few studies reported such information. So, we used Morris formula to calculate SMD based on pre-test and post-test means and pre-test standard deviations, which gives more precise estimates from ESs than common formula based on post-test scores.^31^ Hedges correction for small studies applied to all ESs.

*Addressing multi-arm trials*

In multi-arm trials that compare two or more experimental arms to one shared control group, a somehow common mistake is to repeat the control group in the analysis to compare with each treatment arm. This measure may put excessive weight on these trials (by double-counting the control) and ignore the correlation between ESs. To deal with this issue, we combined the experimental arms (as recommended by the Cochrane^32^). But, this approach does not allow to conduct subgroup analysis based on treatment type. Therefore, we used another solution—to split the shared control group.

*Synthesis method*

Meta-analysis was conducted using a random-effect model with restricted maximum likelihood, as the estimator of true heterogeneity, which seems to provide us with less biased estimates than other methods.^33^

*Additional analyses*

For each analysis, we checked for potential outliers (based on studentized residuals) and influential cases (based on an index analogous to cook’s distance in meta-analytic context^34^). We did not conduct any analysis to detect publication bias. Because fail safe-N has several problems and is not recommended to be used. Also, using funnel plot and the related tests are appropriate only if there were considerable differences between trials in terms of their sample size, and at least some of them have large samples which raise their publication chance, even if their results are not statistically significant. This logical basis was not true in the context of our study (for more details on these methods, see Jin et al^35^). Subgroup analyses were conducted based on three pre-specified factors: treatment type, researcher’s education (Ph.D. or M.A.), and pain type.

*Statistical software*

All analyses were conducted in R using the package “metafor”,^36^ except for calculating SMD from ANCOVA which was conducted in the package “compute.es”.^37^

## Results

Our search and screening resulted in 30 eligible trials, including 35 experimental groups, reported in 54 papers/theses (flow diagram of the selection process is presented in Figure 1). The total sample size was 1021 (with an average of 34 participants per trial). The most common condition across trials was irritable bowel syndrome (IBS) (43%), followed by low back pain (n = 7 [23%]). Female patients constituted 86% of the total sample, with 18 trials conducted only on female patients (60% of trials). One trial was conducted on male patients, and the others included participants from both men and women. The majority of trials used either cognitive-behavioral or third wave treatments. Twelve trials conducted follow up assessment with an average period of 8.6 weeks. The characteristics of the included studies are presented in Table 1.

### Risk of bias in included trials

The risk of bias due to randomization processes (i.e., sequence generation and allocation concealment) was high or unclear in all trials with one exception. Half of the trials were at high or unclear risk of incomplete cases, and less than half were in selective outcome reporting (see Table 1).

### Depression

**Figure 2:**
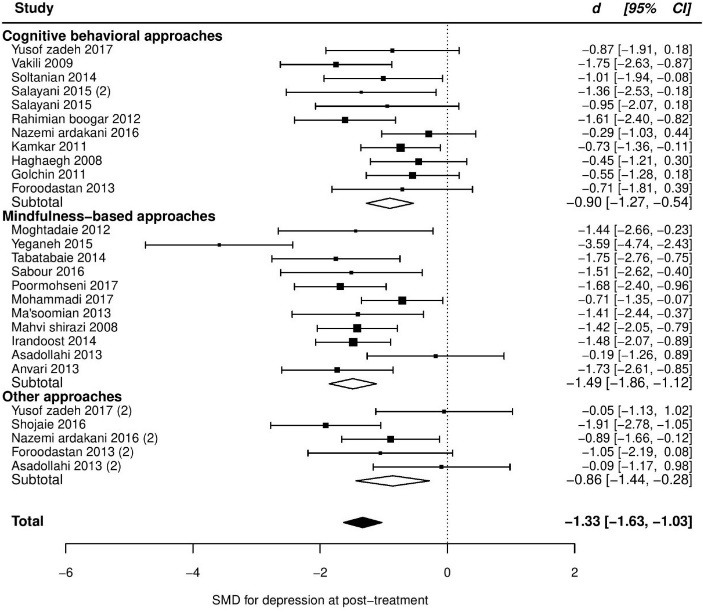
Forest plot of group differences in depression at post-treatment

At post-treatment, pooled SMD from 23 trials was -1.42 (95% CI: -1.76 to -1.09), with significant heterogeneity between trials (*τ*^2^ = 0.47, *Q*_(df = 22)_ = 80.1, *P*_Q_ < 0.001, I^2^ = 74%). Additional investigation detected one outlier with an unexpectedly huge ES (*d* = -6.18, studentized deleted residual = -3.64). This study had huge ESs in anxiety, as well (Cohen’s *d* in anxiety was more than 3). So, we removed it from further analyses in all outcomes. The removing of this study resulted in a slight reduction in ES (SMD = -1.33), but heterogeneity remained significant (*τ*^2^ = 0.34, *Q*_(df = 22)_ = 62.8, *P*_Q_ < 0.001, I^2^ = 68%). Among our pre-specified potential moderators, intervention type significantly moderated the effect, explaining 22% of the heterogeneity (Table 2), but the residual heterogeneity was still significant (*P*_Q_ < 0.05). More details about subgroup analyses are presented in Table 2 and the forest plot of individual ESs within each study are presented in Figure 2.

**Figure 3:**
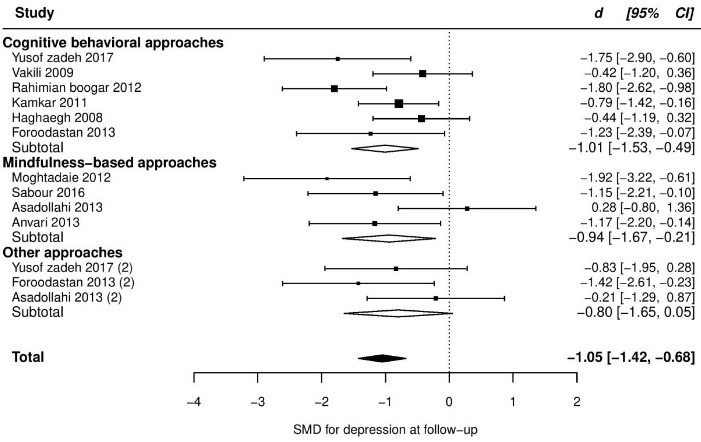
Forest plot of group differences in depression at follow-up.

At follow up, pooled SMD from 10 trials was -1.05 (95% CI: -1.42, -0.68), with significant heterogeneity between trials (*τ*^2^ = 0.17, *Q*_(df = 10)_ = 17.6, *P*_Q_ < 0.04, I^2^ = 49%). One study had a positive ES (*d* = +0.06, indicating negative effect on depression; studentized deleted residual = +2.69). After removing this study, ES increased slightly (SMD = -1.16) and heterogeneity reduced to a non-significant level (*τ*^2^ = 0.03, *Q*_(df = 9)_ = 8.7, *P*_Q_ < 0.35, I^2^ = 16%). However, the data point seemed to be valid, and though it may not be a good idea to be deleted, unless as a part of sensitivity analysis. So, we did not remove this study from other analyses. Retaining this study in the model and adding pain type as a moderator also explained almost all observed heterogeneity. Given this subgroup analysis, the patients with musculoskeletal pain were found to significantly experience more improvement than the patients with IBS. More details are presented in Table 2 and the forest plot of individual ESs is presented in Figure 3.

### Anxiety

**Figure 4:**
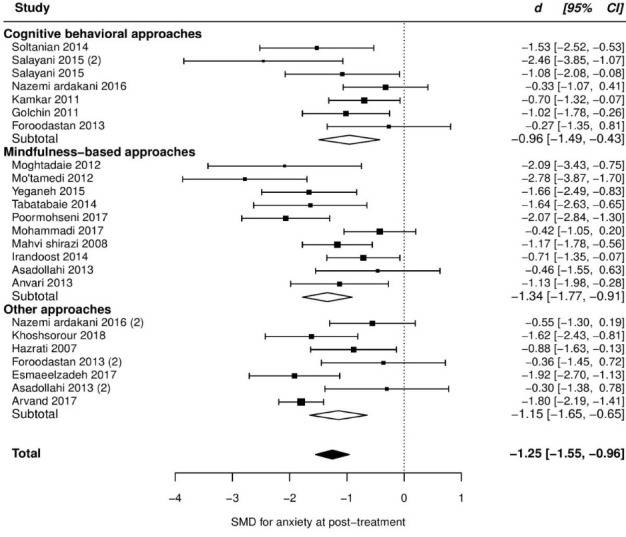
Forest plot of group differences in anxiety at post-treatment.

Twenty trials reported post score on anxiety, which resulted in a large and significant, but very heterogeneous ES (SMD = -1.55, 95% CI [-2.13 to -0.97], τ^2^ = 1.64, Q_(df = 20)_ = 122.8, *P*_Q_ < 0.001, I^2^ = 92%). Additional investigation revealed an outlier with an unusual effect and high influence on the fitted model (*d* = -8.55, studentized deleted residual = -6.65, cook’s *d* = 1.00). After removing this study, we found a slight reduction in the pooled ES (SMD = -1.25, 95% CI, -1.55 to -0.96), but heterogeneity remained moderate and highly significant (*τ*^2^ = 0.3, Q_(df = 19)_ = 62.1, *P*_Q_ < 0.001, I^2^ = 69%). Between our pre-specified moderators, pain type showed a marginally significant moderation effect explaining 19% of the variation. But this moderation effect was, in fact, a function of one category containing one study with a high ES (see Table 2), even though residual heterogeneity was still highly significant (*P*_Q_ < 001). More details are presented in Table 2 and the forest plot of individual ESs is presented in Figure 4.

**Figure 5:**
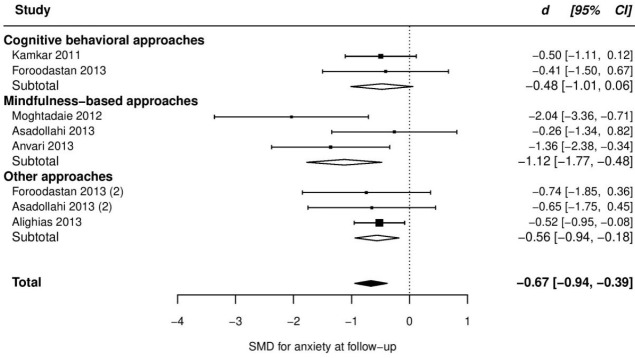
Forest plot of group differences in anxiety at follow-up.

At follow up, pooled SMD from 7 trials was very large and significant (SMD = -1.34, 95% CI [-2.32 to -0.36]), with very high heterogeneity between ESs (τ^2^ = 1.52, Q_(df = 6)_ = 37.2, *P*_Q_ < 0.001, I^2^ = 91%), which was due to an outlier with an unusual ES (d = -4.3, studentized deleted residual = -5.34). After removing this data-point, pooled ES dropped to one half (SMD = -0.67, -0.94, -0.39), and heterogeneity declined to a non-significant level (τ^2^ < 0.01, Q_(df = 5)_ = 8.6, *P*_Q_ = 0.13, I^2^ = 0%). Comparing our follow up to post-treatment ES, we observed a substantial decline in summary effect. Such a decline may be merely due to the possibility that the studies conducted follow up assessment, in general, reported lower ESs, not only at follow up but also at post-treatment. To test this hypothesis, we ran a meta-analysis on the post-treatment scores of the trials with follow up assessment. Results showed these trials with smaller pooled ES at post-treatment as well (SMD at post-treatment = -0.74, 95% CI [-1.12 to -0.36])- approximately equal to their follow up ES. More details are presented in Table 2 and the forest plot of individual ESs is presented in Figure 5.

## Discussion

Our results suggested that psychological interventions improve both depression and anxiety in Iranian patients with CP, with approximately equal and large ESs. These effects, however, seem to vary across situations and may decline slightly over the time of post-interventions. Subgroup analyses revealed a possible superiority of mindfulness-based treatments over other approaches. Also, patients with musculoskeletal pain may respond better to psychotherapies than patients with IBS, though both groups gain large benefits from these treatments. Other subgroup analyses, based on the researcher’s education level, suggested that the trials conducted by MA researchers, compared to the other interventionists, might result in better effects. It is noteworthy that the results of subgroup analyses should be considered as exploratory findings,^29,38^ which need to be tested directly in original studies. Also, generalizing our results to male patients might be questionable, as they comprised only about 15% of our total sample size. Although, given this gender distribution, we could be confident in the efficacy of psychotherapies in women.

Comparing our results to those reported in previous meta-analyses (which were mainly conducted in the United States and Europe) we identified substantial differences in the ESs, both in depression and anxiety. Those meta-analyses mostly reported small to medium-sized effects for psychological interventions on depression, and almost the same on anxiety (ESs from 8 previous meta-analyses were presented in Table 3). However, in our included trials the estimated ESs were large, both for depression and anxiety.

These differences could be attributable to at least three possible reasons:

*First* , differences among populations in pain perception and its related constructs and also their reactions to psychotherapies could affect therapeutic outcomes. However, investigating these factors is beyond the scope of our study, and requires cross-cultural investigations. This factor can moderate the true underlying ES of a given intervention on different populations.

*Second*, differences in the characteristics of included trials could have also changed the magnitude of the observed effects. Obviously, this factor moderates the estimated ES only, and not the true underlying effect. Several well-known factors could elevate the ES. In Table 3, we compared our included trials to the trials included in previous meta-analyses in terms of these factors. The randomization process (including random sequence generation and allocation concealment) may be noticed as an example. Empirical evidence suggests that the trials with adequate randomization tend to produce smaller ESs,^28^ particularly in subjective outcomes,^39^ which is the case in our study. In the included trials of our study, only one trial (3%) had low risk of bias due to randomization. Sample size may be considered as another factor. The correlation between sample size and ES was estimated to be as high as -0.52 in tests of mean difference,^40^ indicating that studies with larger samples produce smaller ESs. The most of trials included in our meta-analysis had relatively small sample sizes (on average 34 participants per trial), which are much smaller than those reported in the trials included in previous meta-analyses (see Table 3).

*Third* , cultural differences may affect the results of a trial, not due to the population’s differences in response to treatment, but as a result of the sampling process of the trial. Among cultures with a common bias towards psychotherapies, or populations who view pain as a purely physical phenomenon, the participants who join psychotherapy trials may have special characteristics, such as openness. In fact, research samples in pain trials in some cultures may not be a representative of the patients with CP in that culture. So, we may consider this factor as *culture’s effect on the external validity* of trials.

However, we also found considerable differences in the trials’ characteristics between Iranian and non-Iranian studies (mainly from the United States and Europe).It is difficult to assume that these factors are responsible for all the observed differences between the ESs. Of course, it is possible that the underlying ES to be truly different. Consequently, if the Iranian CP population or other similar populations (e.g., most countries in the Middle East) respond better to psychological treatments, it would be a great opportunity for improving health, particularly in such a pervasive health problem. To that end, conducting large trials with rigorous methodology and clear reports (which facilitate replication) is probably the first step.

## Conclusion

Our results suggested that psychological interventions are highly effective in reducing anxiety and depression among Iranian patients with CP; and the effects were observed to be persistent for at least several months. Acceptance and mindfulness­­–based treatments may be more effective than other approaches. Also, patients with musculoskeletal pain may gain more benefits from these treatments than patients with IBS.

Comparing our results to other meta-analytical findings, we identified considerable differences in ESs, which led us to the most important question raised from this research: Do these differences related to cultural variations or they are only the functions of trials’ quality? Although we cannot rule out possible effects of culture, our investigations pointed out to some possible methodological factors which may have elevated the pooled ESs- factors like high risk of bias and small sample sizes. Modifications in the quality of RCTs seem to be necessary to confirm the differences in true ES; and if so, it might be a great opportunity for health care systems in Iran and other similar countries.

## Ethical approval

Not applicable.

## Competing interests

The authors declared no conflict of interest.

## Funding

This research received no financial support.

## Authors’ contributions

GF: study design, manuscript revision; MF: study design, manuscript revision; AZ: study design, data collection, statistical analysis and interpretation, and manuscript writing.

## Acknowledgment

We wish to thank the anonymous reviewers for their constructive comments.

**Table 1:** Characteristics and risk of bias for each included trial

**Study**	**References**	**Follow up (week)**	**Pain type**	**Intervention (delivery, sessions)**	**Outcome measures** ^a^	**Sample size**	**Female %**	**Risk of bias**			
								**Sequence generation**	**Allocation concealment**	**Incomplete data**	**Selective report**
Alighias 2013	^41,42^	12	LBP	Psychological education (group, 4)	DASS-21	84	100%	?	?	√	√
Anvari 2013	^43^	8	CP	ACT (group, 8)	DASS-21	17	0%	?	?	×	√
Arvand 2017	^44^	-	IBS	Music therapy (group, 10)	BAI	69	100%	?	?	?	?
Asadollahi 2013	^45^	8	IBS	(1) MBCT (group, 8) (2) spirituality therapy (group, 8)	SCL-90	30	100%	?	?	?	√
Esmaeelzadeh 2017	^46,47^	-	IBS	Emotional intelligence training (group, 12)	BAI	36	Unclear	?	?	√	?
Adarvishi 2016	^48^	-	IBS	Problem solving training (group, 8)	STAI^b^	46	65%	?	?	√	?
Foroodastan 2013	^49^	-	MSP	(1) CBT (individual, 8) (2) quality of life therapy (individual, 8)	DASS-42	30	100%	?	?	√	×
Poormohseni 2017	^50^	-	IBS	MBCT (group, 8)	GHQ-28	40	65%	?	?	√	?
Golchin 2011	^51,52^	-	LBP	CBT (group, 12)	DASS-21	30	100%	?	?	√	√
Hazrati 2007	^53,54^	12	IBS	Bensons' relaxation (audio tape, 3 months daily)	STAI	30	73%	×	?	×	?
Haghaegh 2008	^55^	8	IBS	CBT (group, 8)	BDI	28	58%	?	?	×	√
Irandoost 2014	^56^	8	LBP	ACT (group, 8)	HADS CES-D	40	100%	?	?	√	×
Jokar 2009	^57^	4	RA	Cognitive behavioral stress management (group, 10)	DASS-42	16	100%	?	?	×	√
Khoshsorour 2018	^58^	-	IBS	Neurofeedback (individual, 10)	STAI	30	100%	?	?	√	?
Soltanian 2014	^59^	-	RA	CBT (group, 8)	DASS-21	20	100%	?	?	?	√
Sabour 2016	^60^	4	CP	ACT (group, 8)	DASS-21	16	100%	?	?	×	×
Tabatabaie 2014	^61^	-	IBS	Meta cognitive therapy (group, 8)	HADS	21	100%	?	?	√	√
Kamkar 2011	^62^	12	IBS	Cognitive behavioral stress management (group, 8)	BAI	42	48%	?	?	?	√
Ma'soomian 2013	^63,64^	-	LBP	MBSR (unclear, 8)	DASS-21	18	100%	?	?	×	×
Rahimian Boogar 2012	^65^	16	LBP	CBT+pain management (group, 8)	DASS-42	35	48%	√	√	?	√
Salayani 2015	^66,67^	3	CP	(1) CBT (group, 8) (2) CBT+pain management (group, 8)	DASS-21	30	100%	?	?	√	√
Shojaie 2016	^68^	-	CP	The transtheoretical model (stages of change) (group, 8)	BDI	30	100%	?	?	√	√
Mohammadi 2017	^69^	-	IBS	MBCT (group, 8)	BAI	40	Unclear	?	?	√	√
Mahvi shirazi 2008	^70,71^	-	IBS	CBT (unclear, 8)	SCL-90	50	59%	?	?	√	√
Vakili 2009	^72,73^	8	LBP	CBT (group, 8)	SCL-90	26	100%	√	?	?	√
Yeganeh 2015	^74^	-	RA and lupus	MBSR (group, 8)	DASS-21	30	100%	?	?	√	√
Yusof Zadeh 2017	^75,76^	8	LBP	(1) CBT (individual, 6) (2) schema therapy (individual, 6)	DASS-21	35	74%	√	?	×	?
Mo'tamedi 2012	^77^	-	Headache	ACT (group, 8)	STAI-trait	26	100%	?	?	×	×
Moghtadaie 2012	^78,79^	8	IBS	MBCT (group, 8)	BSI	14	100%	?	?	×	√
Nazemi Ardakani 2016	^80,81^	-	RA	(1) Cognitive behavioral stress management (group, 8) (2) Islamic spiritual therapy (group, 8)	DASS-21	62	Mostly	?	?	√	√

Abbreviations: LBP = low back pain; CP = chronic pain; IBS = irritable bowel syndrome; MSP = musculoskeletal pain; RA = rheumatoid arthritis; ACT = acceptance and commitment therapy; MBCT = mindfulness-based cognitive therapy; CBT = cognitive behavioral therapy; MBSR = mindfulness-based stress reduction; DASS = depression, anxiety, and stress scale; BAI = Beck anxiety inventory; SCL = symptom check list; STAI = state-trait anxiety inventory; GHQ = general health questionnaire; BDI= Beck depression inventory; HADS = hospital anxiety and depression scale; BSI = brief symptom inventory. ^a^ It only includes outcome measures used in this meta-analysis. In multifactor scales (e.g., SCL-90, GHQ-28), only scores from anxiety and/or depression subscales were used. ^b^ In this measure, we used the state subscale, as it is sensitive to change, and which is comparable to other anxiety scales. √ = low risk of bias; O = high risk of bias; ? = unclear risk of bias.

**Table 2:** Summary of meta-analytical results and subgroup analyses

	**Factor**	**Condition**	**k**	**N**	**SMD**	**95% CI**	**Test for moderator**
Depression (Post-treatment)		**Total**	**22**	**684**	**-1.33**	**-1.64, -1.03**	**-**
	Treatment type	Cognitive behavioral	11	277	-0.90	-1.27, -0.54	*Q* (*df* = 2) = 5.84, *P* = 0.05, *R*^2^ = 23%
		Mindfulness-based	11	301	-1.49	-1.86, -1.12	
		Others	5	106	-0.86	-1.44, -0.28	
	Pain type	MSC	15	419	-1.52	-1.89, -1.15	*Q* (*df* = 1) = 2.55, *P* = 0.11, *R*^2^ = 9%
		IBS	7	265	-1.02	-1.50, -0.55	
	Researchers education	MA	14	350	-1.46	-1.85, -1.07	*Q* (*df* = 1) = 0.98, *P* = 0.32, *R*^2^ = 0%
		PHD	8	334	-1.15	-1.62, -0.67	
Depression (follow up)		**Total**	**10**	**273**	**-1.05**	**-1.42, -0.68**	**-**
	Treatment type	Cognitive behavioral	6	166	-1.01	-1.53, -0.49	*Q* (*df* = 2) = 0.17, *P* = 0.92, *R*^2^ = 0%
		Mindfulness-based	4	62	-0.94	-1.67, -0.21	
		Others	3	45	-0.80	-1.65, +0.05	
	Pain type	MSP	6	159	-1.35	-1.70, -1.00	*Q* (*df = 1* ) = 8.6, *P* = 0.003, *R*^2^ = 100%
		IBS	4	114	-0.57	-0.96, -0.18	
		MA	6	131	-0.85	-1.33, -0.38	*Q* (*df* = 1) = 1.43, *P* = 0.23, *R*^2^ = 15%
		PHD	4	142	-1.29	-1.82, -0.76	
Anxiety (post-treatment)		**Total**	**20**	**687**	**-1.25**	**-1.55, -0.96**	**-**
	Treatment type	Cognitive behavioral	7	168	-0.96	-1.49, -0.43	*Q* (*df* = 2) = 1.23, *P* = 0.54, *R*^2^ = 0%
		Mindfulness-based	10	293	-1.34	-1.77, -0.91	
		Others	7	226	-1.15	-1.65, -0.65	
	Pain type	MSP	8	259	-1.04	-1.48, -0.61	*Q* (*df* = 2) = 5.18, *P* = 0.07, *R*^2^ = 19%
		IBS	11	402	-1.29	-1.66, -0.92		
		Headache	1	26	-2.78	-4.24, -1.33	
	Researchers education	MA	13	397	-1.39	-1.75, -1.02	*Q* (*df* = 1) = 1.35, *P* = 0.24, *R*^2^ = 7%
		PHD	7	360	-1.03	-1.50, -0.55	
Anxiety (follow up)		**Total**	**6**	**217**	**-0.67**	**-0.94, -0.39**	
	Treatment type	Cognitive behavioral	2	57	-0.48	-1.01, +0.06	*Q* (*df* = 2) = 2.69, *P* = 0.26, *R*^2^ = 0%
		Mindfulness-based	3	46	-1.13	-1.77, -0.47	
		Others	3	114	-0.56	-0.94, -0.18	
	Pain type	MSP	3	131	-0.80	-1.42, -0.17	*Q* (*df* = 1) = 0.01, *P* = 0.95, R^2^ = 0%
		IBS	3	86	-0.77	-1.45, -0.09	
	Researchers education	MA	4	145	-0.92	-1.50, -0.35	*Q* (*df* = 1) = 0.63, *P* = 0.43, *R*^2^ = 0%
		PHD	2	72	-0.54	-1.29, +0.20	

Abbreviations: MSP = musculoskeletal pain; IBS = irritable bowel syndrome.

**Table 3:** Reported effect sizes in previous non-Iranian meta-analyses and possible characteristics which might explain the difference

**Study (Reference)**	**SMD for depression:** **Post-treatment (follow up)**	**SMD for anxiety: Post-treatment (follow up)**	**Characteristics which might contribute in the magnitude of SMDs**
Current study	-1.33 (-1.05)	-1.25 (-0.67)	Trials with adequate randomization = 3% Mean sample across included trials = 34
Morley et al 1999 ^16^	-0.36	-	Trials with clear randomization details = 16% Mean of the sample size across included trials = 57 Objective outcomes (not self-reports) = 23%
Williams et al 2012^17^	CBT: -0.38 (-0.26) BT: -0.53 (-0.65)	-	Trials with adequate randomization = 24% Mean sample across included trials = 114
Hoffman et al 2007^18^	-0.31	-	Mean sample = 79 Exclusive to chronic low back pain
Veehof et at 2016^19^	-0.43 (-0.50)	-0.51 (-0.57)	Mean sample = 51 Only mindfulness and acceptance-based treatments
Hilton et al 2016^20^	-0.15	-	Mean sample = 92 Only mindfulness-based treatments
Bernardy et al 2017^21^	-0.43 (-0.48)	-	28% with low risk in randomization Mean sample = 86 Only CBT for fibromyalgia
Haugmark et al 2019^82^	-0.49 (-0.48)	-0.37 (-0.34)	44% with low risk for randomization Mean sample = 83 Only mindfulness and acceptance-based treatments for fibromyalgia
Dixon et al 2007^83^	-0.21	-0.28	Mean sample = 126 Only patients with arthritis

Abbreviations: CBT = cognitive behavioral therapy, BT = behavioral therapy.
